# A simple breathing circuit allowing precise control of inspiratory gases for experimental respiratory manipulations

**DOI:** 10.1186/1756-0500-7-235

**Published:** 2014-04-12

**Authors:** Felipe B Tancredi, Isabelle Lajoie, Richard D Hoge

**Affiliations:** 1 Institut de génie biomédical, Département de physiologie, Université de Montréal, C.P. 6128, Succursale Centre-ville, Montréal, Québec H3C 3J7, Canada; 2 Unité de neuroimagerie fonctionnelle, Centre de recherche de l’institut universitaire de gériatrie de Montréal, Montreal, QC, Canada; 3 Centre de recherche de l’institut universitaire de gériatrie de Montréal, 4545, Queen Mary, Montreal, QC H3W 1W5, Canada

**Keywords:** Respiratory manipulation, Functional MRI, Hypercapnia, Hyperoxia, Breathing circuit

## Abstract

**Background:**

Respiratory manipulations modulating blood flow and oxygenation levels have become an important component of modern functional MRI applications. Manipulations often consist of temporarily switching inspired fractions of CO_2_ and O_2_; and have typically been performed using simple oxygen masks intended for applications in respiratory therapy. However, precise control of inspired gas composition is difficult using this type of mask due to entrainment of room air and resultant dilution of inspired gases. We aimed at developing a gas delivery apparatus allowing improved control over the fractional concentration of inspired gases, to be used in brain fMRI studies.

**Findings:**

The breathing circuit we have conceived allowed well controlled step changes in FiO_2_ and FiCO_2_, at moderate flow rates achievable on standard clinical flow regulators. In a two run test inside the scanner we demonstrate that tightly controlled simple gas switching manipulations can afford good intra-subject reproducibility of induced hyperoxia/hypercapnia responses. Although our approach requires a non-vented mask fitting closely to the subject’s face, the circuit ensures a continuous supply of breathable air even if the supply of medical gases is interrupted, and is easily removable in case of an emergency. The apparatus we propose is also compact and MRI compatible, allowing subject placement in confined spaces such as an MRI scanner for brain examinations.

**Conclusions:**

We have reported a new approach for the controlled administration of medical gases, and describe an implementation of the breathing circuit that is MRI compatible and uses commercially available parts. The resultant apparatus allows simple, safe and precise manipulations of FiO_2_ and FiCO_2_.

## Background

**Figure 1:**
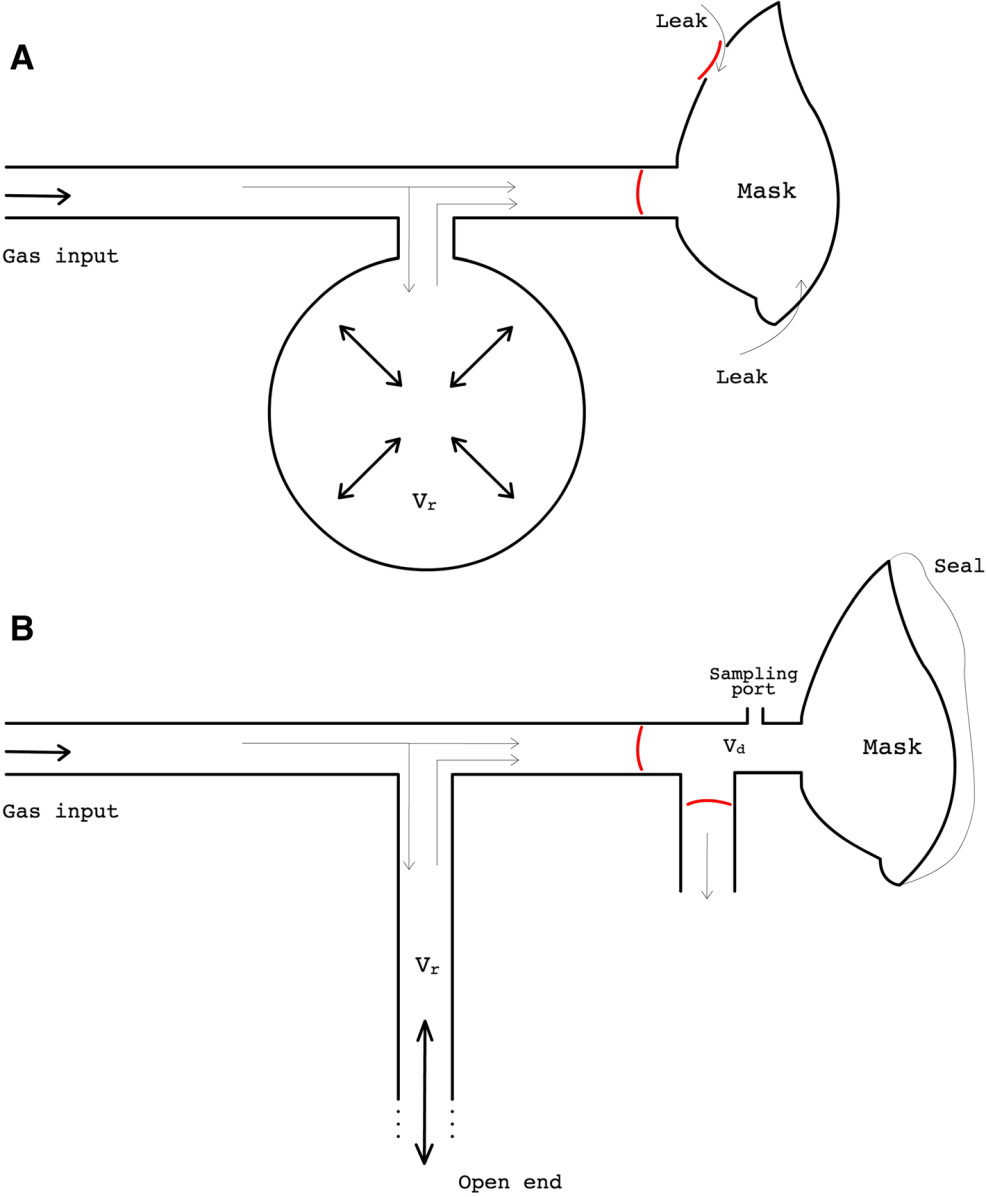
**Schematic comparison between the new breathing circuit and a non-rebreathing oxygen mask.** A typical oxygen mask/circuit is shown on the top **(A)**. The input gas may go straight to the mask or fill the breathing bag when delivery rate exceeds uptake rate (e.g. during the expiratory phase of the breathing cycle). Whenever uptake exceeds delivery rate the bag empties to provide supplementary gas for inspiration. Expired gases leave the mask through vents controlled by valves. Gases inside the mask are often contaminated by air that leak in through the edges of the mask and/or through the vents when valves are malfunctioning. This compromises control over the inspiratory gases as well as the accurate monitoring of end-tidal values. To avoid the latter concerns the new breathing circuit we have developed **(B)** uses a non-vented mask providing a sealed fit to the subject’s face. But also, the breathing bag is replaced by a long limb reservoir with an open end, which makes the circuit completely safe as the person can normally breathe room air if no gas is supplied. Besides, the geometry of such reservoir permits faster transitions in FiO_2_ and FiCO_2_. Expired gases are exhausted through a second limb, controlled by an efficient unidirectional valve. Lastly, a sampling port is installed adjoined to the mask to help the respiratory monitoring. Vr = volume of the reservoir; Vd = dead space added by appending the dual limb system to the mask.

Respiratory manipulations modulating the fractional concentration of inspired O_2_ and CO_2_ (FiO_2_ and FiCO_2_) to induce hyperoxia/hypercapnia and modulate cerebral blood flow and oxygenation have become an important component of fMRI studies measuring the vascular as well as the metabolic function in the brain [1-7]. Methods using computerized systems to control modulations in inspired concentrations and target specific end-tidal levels have been proven successful in enabling inter-subject reproducibility of hyperoxic/hypercapnic stimuli [8-13] as well as in providing flexibility in the achievable gas mixtures [14-16] to test different hypotheses. While individualized mixing of gas concentrations to achieve prospective control of end-tidal levels is useful in certain situations, the requirements for many applications are met by delivering predetermined concentrations of inspired gases, so long as end-tidal values are recorded for retrospective normalization of the fMRI signal. The latter, simpler, approach thus continues to be widely adopted in quantitative neuroimaging and other areas. A common practice has been to administer fixed fractional concentrations of O_2_ and CO_2_ for inhalation [3,5,6,17], delivering O_2_/CO_2_ enriched mixtures through low-cost nonrebreathing masks commonly used in clinical oxygen therapy, which usually incorporate a reservoir bag to increase dosage efficiency (the bag reservoir stores the gases delivered during expiration to make it available for next inspiration(s), making better use of administered gases and alleviating dropouts in FiO_2_ and FiCO_2_ during early phases of inspiration) [18]. However, either due to variations in the shape of the subject’s face or to incomplete sealing of one-way valves used to release excess gas flow, such oxygen masks are often very leaky [18], hampering precise adjustments in fractional inspired concentrations and thus limiting the reproducibility of the hyperoxic/hypercapnic stimuli. One way around this limitation would be to ensure a tight seal of the face mask using surgical tape or other means. However, in a closed circuit where the gas supply is directly connected to the mask, this creates a risk of asphyxia in the event that the medical gas supply is interrupted, although this can be mitigated through the incorporation of special safety measures. To improve control over inspired gases we have conceived a breathing circuit whose different compartments are separated by high efficiency one-way valves and whose breathing chamber consists of a non-vented mask providing a tight facial fit, but that can rapidly removed. To ensure the safety of subjects, we have replaced the breathing bag found in oxygen masks by an open reservoir, through which the subject breathes room air whenever the flow of administered gases becomes insufficient. The resulting open breathing circuit (Figure 1) represents an inherently safe design and allows precisely controlled step changes in the fractional concentration of inspired gases, with moderate flow rates.

## Findings

### Material and methods

**Figure 2:**
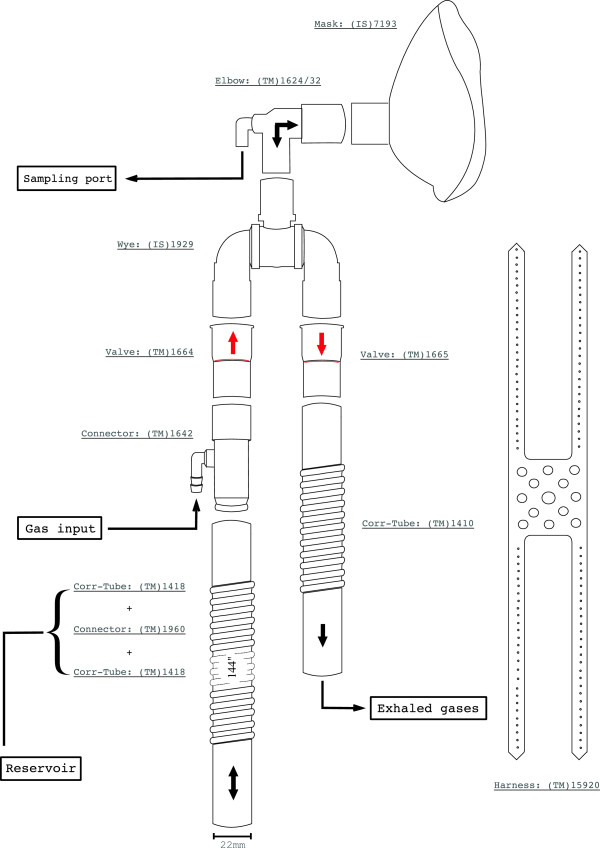
**Schematic of the circuit assembly.** Gas mixtures are supplied through the barbed inlet of the connector TM #1642. The elbow can be of two different types, with a luer-lock or barbed sampling port (TM #1624 or #1632 respectively). The limb reservoir is formed by two corrugated tubings (TM #1418) coupled by a connector (TM #1960), which is not represented here. The mask (IS #7193) comes with a support (not represented) allowing to attach the harness (TM# 15920).

The breathing circuit we describe below (Figure 2) has been implemented, using commercially available components, to address three design criteria: MRI-compatibility, accommodation within MRI radiofrequency coils enveloping the head for brain exams, and sufficiently low cost to justify disposal after use to avoid transmission of airborne or other pathogens. Parts were acquired from two different vendors: Intersurgical Inc (NY, USA) and Teleflex Medical (NC, USA); which we will be referred to as IS and TM respectively.

The circuit comprises a small non-vented face mask (IS #7193) and a dual-limb airway that is appended to the mask’s frontal opening. An elbow (TM #1632 or #1624) and a triple swivel wye-piece (IS #1929) connect the limbs to the mask. A pair of valves (TM #1664/5) at the join of the limbs ensure that 1) inspired gases only come from the incoming limb, consisting of a corrugated tube (2 TM #1418 connected by TM #1960) that is preceded by a connector (TM #1642); and 2) that expired gases only flow through the outgoing limb, that can be a short corrugated tube (TM #1410). These two limbs have an open end, communicating with the exterior. While the small outgoing limb serves as an exit for expired gases, the long incoming limb serves as a gas reservoir, like the breathing bag of conventional oxygen masks. However, because of its geometry (we used ~3.5 m of corrugated tube with 22 mm internal diameter) and open end, the limb reservoir functions as a sequential container, where administered gases are stacked as they arrive at the circuit and can be expected to mix less than in a bag type of reservoir. This way, during transitions between gas mixtures of different concentrations, the new mixture becomes readily available for inspiration, allowing sharp transitions in fractional concentration of inspired gases. Whereas the same effect could be achieved without this long limb reservoir, that would require high flow dosages to meet peak inspiratory rates.

The circuit was conceived to allow better isolation between circulating gases and exterior, while ensuring a constant supply of breathable gas even in the event of an interruption in the gas administration. The anesthetic mask we have adopted offers a tight facial fit, drastically reducing entrainment of room air and resultant dilution of inspired/expired gases (as can occur with Hudson-style oxygen masks) but can be very easily removed in case of danger or discomfort. The mask does not need to be sealed using adhesive tape [8] nor held to the head using restrictive harnesses that can be difficult to remove in an emergency [19] (we have adopted the TM #15920). More importantly though, as mentioned above, the gas reservoir of our circuit has an open end which protects the subject against disruptions in the gas supply. Whenever the gas administration is insufficient to meet the subject’s ventilation the reservoir is replenished with room air for inspiration, i.e. there is no special requirements to ensure a constant supply of breathable air.

The proposed circuit is also equipped with a sampling port for the monitoring of O_2_/CO_2_ and pressure wave inside the mask chamber, avoiding the use a nasal cannula and potential discomfort associated with this. The sampling port is positioned at the elbow that connects the mask to the circuit’s limbs and can be of two types, barbed (elbow TM #1632) or luer lock (elbow TM #1624), depending on the tubing used for the sampling line.

As a proof of concept of the improvement in respiratory manipulations attained with the new circuit we have conducted two tests using both the new circuit and a non-vented Hudson oxygen mask (#1060) in a young healthy subject (female: 32y, 1.65 m, 60 kg). In Test 1 the subject was given 100% O_2_ or 50% O_2_ balanced with air in two different instances lasting 3 minutes each and 3 minutes apart. Medical air was administered otherwise. Flow rates were 15 L/min during the whole manipulation. Test 2 followed the same design as Test 1 but with different flow rates. Upon transitions in administered concentrations, flow rates were increased to 30 L/min for 1 m:30s.

The new circuit has been deployed in multi-subject fMRI experiments conducted by our group, in which FiO_2_ and FiCO_2_ were modulated administering 100% O_2_ and 5% CO_2_ according to the schedule proposed by Bulte et al. [7]. The circuit has been used in over 20 MRI sessions, with very consistent results. We will show here data obtained from single representative participant who underwent two different runs of the above protocol.

Respiratory gases were monitored using Biopac MP150 (Biopac Systems Inc., CA, USA). Fractional concentrations of O_2_ and CO_2_ were analyzed and recorded at a 50 ms sampling rate. The gas sampling line consisted of a long segment of rigid tubing pertaining to the monitoring equipment (AFT31-XL) preceded by a disposable bacterial filter (#2200/01, Air Safety Medical Ltd,) and a short segment of oxygen tubing (#2001, Salter Labs, CA, USA) to connect the filter to the sampling port of our breathing circuit (built using a TM #1632 elbow). We noted that, using the current setup, accurate measures of fractional concentration were difficult to obtain when breathing rates exceeded 10 breaths per minute. We believe this effect is mainly associated with the limited rise/fall time of our gas analyzers and mixing of gases inside the sampling line; but we have also observed that the bacterial filter placed in the sampling line contributes to the problem, although we are not certain through which exact mechanisms. In future experiments we intend to 1) replace the long segment of the sampling line by a narrow-bore plastic tubing (then, to overcome the resistance it adds to the line, attach an external sampling pump to Biopac) and 2) test other solutions to filter the sampling gases. In the present study, however, to obtain crisp respiratory signal traces we have instructed subjects to limit their respiratory rate to 10 breaths per minute. If the subject breathes at a pace lower than usual there may be less contamination of inspired gases in breathing circuit, which will result in better performance controlling for inspired doses (even if the decrease in respiratory rate is just minor such as in the particular case of the herein experiments). However, the same is true for the oxygen mask, i.e. a reduced respiratory rate also lessens contamination of inspired gases and improves the mask’s performance. Therefore we considered that the special requirement to cap respiratory rate should not invalidate the comparison between performances of the two apparatuses. On the contrary, we considered that it would help better illustrating the differences one should observe if using a robust sampling method.

The pressure wave at the end of the sampling line was monitored using an in-house transducer that connected to one of Biopac’s analog channels. This signal helped identifying the expired (i.e. end-tidal) points in the post processing of O_2_ and CO_2_ respiratory traces.

Fractional concentrations were multiplied by 760 mmHg to be converted in approximate partial pressure values. Baseline levels and changes in end-tidal partial pressures were quantified using the approach described in ref [17]. The end-tidal sampled points were fit to a linear model consisting of a third degree polynomial term plus the CO_2_/O_2_ administration periods as the response regressors, which were shaped using bi-exponential functions to improve the contour of transitions. Baseline levels were obtained from the regressors representing the offset term of the model; respiratory responses represented the effect size of fitted response regressors.

## Results

**Figure 3:**
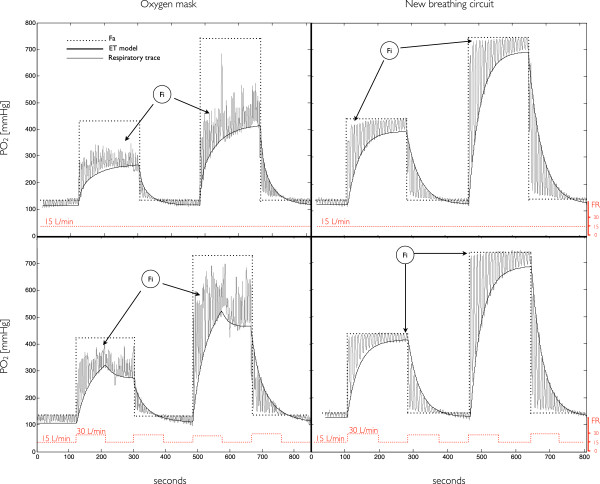
**Respiratory traces of hyperoxic manipulations using the new circuit (right) vs. an oxygen mask (left).** In the first row we show tests where 50% O_2_ and 100% O_2_ were administered in two different instances and in which flow rate was 15 L/min during the entire manipulation. In the second row we repeated the first experiments but increasing the flow rate to 30 L/min during 1 m:30s after transitions. Fractional concentrations are given as partial pressure with respect to an atmospheric pressure of 760 mmHg. FA = fractional concentration of administered O_2_; Fi = fractional concentration of inspired O_2_; ET model = model of the fractional concentration of expired O_2_; FR = flow rate.

In Figure 3 we show results of Tests 1 and 2; tests with the oxygen mask are shown on the left column whereas test with the new circuit on the right.

From the plots on the left column we note how different the composition of actual inspired gases (FiO_2_) and the composition of administered gases (FA) can be when the oxygen mask is used. Furthermore, in the second row we have a clear example of how dependent the composition dosage – i.e. FiO_2_ – can be on the flow dosage – i.e. flow rate (FR). The administration of 100% O_2_ at different FR’s induces different FiO_2_ inputs (as well as end-tidal responses). This is related to the contamination of administered gases by room air that leaks in to the mask: for lower FR’s the pressure inside the mask is lower, which worsens the leak and reduces FiO_2_. Conversely, when fixed FR’s are used (which is often the case) the degree of leak will vary according to the fit of the oxygen mask to the subjects’ face. This compromises inter- but as well intra-subject reproducibility of the FiO_2_ manipulations.

In the tests with the new circuit (right column) there was a very close correspondence between FA and FiO_2_. In the test where FR was kept constant throughout the manipulation (first row) we note that the transitions in FiO_2_ were not as sharp as the switching of gases. This results from the small contamination of new input gases with gases that remained in the limb reservoir from the preceding input phase. In the test where FR is increased from 15 L/min to 30 L/min upon the switching of gases (second row) the replacement of gases from the preceding input by the gases constituting the new FA input is faster, which results in FiO_2_ transitions that are sharper. Such maneuver can be used in other experiments as a means to achieve squared step changes in inspired doses. However, in this particular manipulation, the average FR was increased by 100% of the regular flow rate – the limb reservoir being flooded with approximately 22 L of excess gases during each of the 1 m:30s transitional periods. To keep the average FR close to normal levels and minimize gas usage, the flush procedure should be as fast as possible. Ideally one would deliver a short bolus of the new FA input with the approximate volume of the limb reservoir. Nevertheless, the flush does not need to be well controlled and in fact any increase in flow rate has the effect of accelerating the transitions in inspired doses. For instance, if flow rates must be limited to a maximum of 20 L/min, keeping FR at this level for 20–30 seconds will greatly improve transitions in the inspired doses. Yet, in the many cases where traceability of flow rates is not a concern, the simplest approach to flush the limb reservoir would consist of opening the regulator’s valve widely just before setting the flow rate to normal, traceable, levels.

**Figure 4:**
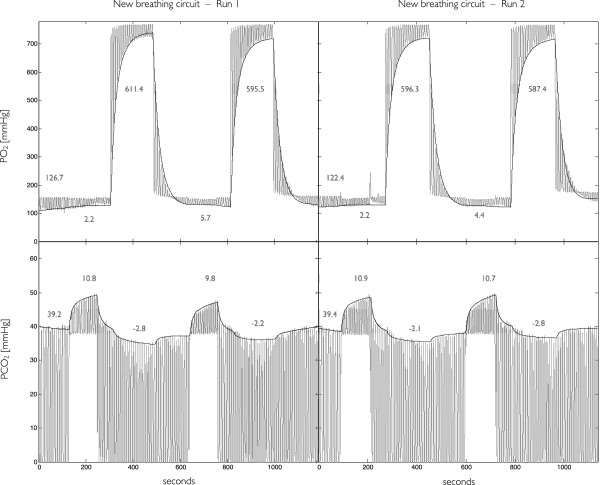
**Reproducibility of O**_**2 **_**and CO**_**2 **_**manipulations using the new breathing circuit.** Manipulations consisted of two runs of the 18-minute schedule described in ref [7]. O_2_ (top) and CO_2_ (bottom) respiratory traces (grey) were modeled as described in Material and Methods to obtain end-tidal (ET) levels (black). Pure O_2_ was administered during 3 minutes in two different instances, inducing an approximate 600 mmHg increase in the fractional concentration of ETO_2_. A 5%CO_2_ mixture was also administered in two different instances, lasting 2 minutes each, which induced an approximate 10 mmHg increase in the fractional concentration of ETCO_2_. We also observed small changes in ETO_2_ and ETCO_2_ when the CO_2_ and, respectively, O_2_ were administered. When not breathing one of the above gas compositions, the subject breathed medical air.

In Figure 4 we show results from manipulations using the circuit in a fMRI experiment for the non-invasive measurement of CMRO_2_[5]. The 18-minute respiratory schedule proposed by Bulte et al. [7], which includes interleaved stimuli of hyperoxia and hypercapnia, was repeated twice. To accelerate transitions in inspired doses, we have momentarily increased FR from 15 L/min to 60 L/min during 3 seconds upon each FA transition, flooding the limb reservoir with 3 L boluses before resumption of the regular flow rate. The change in gas flow during this flushing procedure is greatly attenuated when gases reach the mask (due to dispersion in the long tubes), and is only marginally perceptible to the subject.

The dosage efficiency was again high, as evidenced by the tight correspondence between the fractional composition of the administered mixtures and the fractional composition of the actual inspired gases. In the O_2_ monitoring signal we see the same squared FiO_2_ contours as observed in Figure 3 when the 100% O_2_ is administered; all of which have the same height as the FA of O_2_, i.e. 760 mmHg. In the CO_2_ monitoring signal, we find blank, rectangular areas under the CO_2_ trace when the 5% CO_2_ mixture is administered. The height of these areas correspond well to the FA of CO_2_. Using moderate rates and a simple flushing procedure, our respiratory circuit allowed sharp and precisely controlled step changes in both FiO_2_ and FiCO_2_.

Our test results also demonstrate that end-tidal responses to controlled step changes in inspired concentrations can be quite reproducible. In Figure 4 it can be seen that modulations in end-tidal gas levels are very similar both in terms of their qualitative shape and their amplitude. In each plot, the leftmost value represents the baseline end-tidal level of the respective gas, whereas the 4 other values represent the responses associated to the switching of administered/inspired compositions. Modeled increases in P_ET_O_2_ and P_ET_CO_2_ were both consistent: P_ET_O_2_ increases varied by less than 10% relative to the baseline levels whereas P_ET_CO_2_ increases varied by less than 2%. Since the magnitude of elicited responses to a given challenge depend on the physiological status of the individual, we note that it might not be possible to attain the same degree of intra-subject reproducibility when subjects are scanned in multiple days.

A careful inspection of the O_2_ monitoring trace reveals a small negative FiO_2_ input during the administration of CO_2_. This results from the slightly lower oxygen content in the administered CO_2_ mixture which is composed of 5%CO_2_ and 95% of air. And we also note that in this scheme 1) hyperoxic stimuli may not be isocapnic, i.e. when breathing pure O_2_ the subject’s ETCO_2_ can be markedly depressed; 2) nor the hypercapnic stimuli perfectly iso-oxic as ETO_2_ also changes when the CO_2_ mixture is administered (although this may be less of a concern given the small, < 5%, relative changes in O_2_ levels and its respective impact on the MRI signal).

## Conclusion

We have developed a simple breathing circuit allowing well controlled step changes in the fractional concentration of inspired gases, to be used in applications such as in MRI studies measuring the cerebrovascular responses to respiratory gases. In addition, the circuit ensures uninterrupted availability of gas and operates at moderate flow rates achievable with standard clinical flow regulators.

The type of mask and unidirectional valves we have employed in our circuit, which prevent dilution of administered gases by room air (as can occur when using standard clinical oxygen masks), are the features permitting the control over fractional inspired concentrations. However, the key component of our circuit is the limb reservoir. While optimizing gas consumption, it permits sharper transitions in the fractional inspired concentrations and, more importantly, endows the circuit with a fail-safe mechanism. It should be emphasized that for the proper operation of the circuit and its safety features, the limb reservoir must be kept unobstructed at all times. To ensure total protection of the subject additional safety measures should be included, such as the ongoing and careful monitoring of both inspired and expired respiratory gases, as well as arterial O_2_ saturation.

Although the proposed circuit could also be used in applications entailing frequent adjustments in inspired fractions (e.g. for feed-back control of end-tidal levels) that would require high flow rates to permanently flush the limb reservoir with the right gas composition for inhalation. To control of end-tidal levels at low flow rates alternative methods are warranted [12]. Using the sequential gas delivery method introduced by Banzet et al. [11] – which allows controlling for the effective volume of inspired mixtures – combined with a physiological model of the dependence of end-tidal responses on inspired fractions it is possible to prospectively target end-tidal levels with minimal gas usage (a feed-forward modeling method as opposed to feed-back control). This approach requires a computer controlled mixing system in addition to a specially designed breathing circuit, which is normally taped on the subjects’ face and forms a closed loop (in case of discomfort the subject can remove a safe plug positioned near the nose).

The prototype we have devised using commercially available parts has a dead space that prevented us from having perfectly squared step changes in inspired concentrations. As can be noticed in most transitions, inspired levels in the first breath right after the switch of gases do not attain the administered concentrations. Ideally the circuit should be fabricated using a non-vented and tightly-sealing mask with minimal-size chamber and a gas input as close as possible to the subjects nose/mouth to minimize the dead space. Such a circuit, like the one presented here, should include sampling ports, to avoid the use of nasal cannulas and the potential discomfort associated with these, and be disposable.

In summary, the breathing circuit we have presented 1) allows control over fractional inspired concentrations, 2) optimizes gas consumption in block design manipulations, 3) is simple, 4) safe and 5) comfortable.

## Competing interests

The authors declare that they have no competing interests.

## Authors’ contributions

FBT contributed to the conception and design of the experiments, data collection and analysis, interpretation of the results, and took the lead role in preparation of the manuscript. IL contributed to the data collection, interpretation of results and preparation of the manuscript. RDH contributed to the design of the experiments, interpretation of the results, and preparation of the manuscript. All authors read and approved the final manuscript.
